# Preserving transcriptional stress responses as an anti‐aging strategy

**DOI:** 10.1111/acel.13297

**Published:** 2021-01-20

**Authors:** Yang Cheng, Andrew Pitoniak, Julia Wang, Dirk Bohmann

**Affiliations:** ^1^ Department of Biomedical Genetics University of Rochester Medical Center Rochester New York USA; ^2^ Medical Scientist Training Program Baylor College of Medicine Houston Texas USA; ^3^ Present address: Boehringer Ingelheim Pharmaceuticals Inc Ridgefield Connecticut USA; ^4^ Present address: Jamestown Community College Jamestown New York USA

**Keywords:** aging, chromatin, drosophila, Nrf2, oxidative stress, transcription

## Abstract

The progressively increasing frailty, morbidity and mortality of aging organisms coincides with, and may be causally related to, their waning ability to adapt to environmental perturbations. Transcriptional responses to challenges, such as oxidative stress or pathogens, diminish with age. This effect is manifest in the declining function of the stress responsive transcription factor Nrf2. Protective gene expression programs that are controlled by the Drosophila Nrf2 homolog, CncC, support homeostasis and longevity. Age‐associated chromatin changes make these genes inaccessible to CncC binding and render them inert to signal‐dependent transcriptional activation in old animals. In a previous paper, we have reported that overexpression of the CncC dimerization partner Maf‐S counteracts this degenerative effect and preserves organism fitness. Building on this work, we show here that Maf‐S overexpression prevents loss of chromatin accessibility and maintains gene responsiveness. Moreover, the same outcome, along with an extension of lifespan, can be achieved by inducing CncC target gene expression pharmacologically throughout adult life. Thus, pharmacological or dietary interventions that can preserve stress responsive gene expression may be feasible anti‐aging strategies.

## Graphical abstract

Nrf2‐dependent transcriptional activity declines with age due to epigenetic degeneration at the target promoters in Drosophila. Here, we describe genetic and pharmacological startegies to delay the epigenetic decline and to preserve Nrf2‐target antioxidant gene responsiveness. These findings support the notion that hormetic mechanisms that serve to maintain the function and accessible chromatin structure of signal responsive genes can evoke long‐lasting, anti‐aging effects.

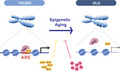

## 1 INTRODUCTION

Organism homeostasis relies on a complex network of cellular sensing and response mechanisms, which maintain the dynamic biochemical equilibria that support life. The robustness of this system declines with progressing age, resulting in an increased susceptibility to disease, decreased tolerance to various types of stress and ultimately higher morbidity and mortality. Aging organisms lose the ability to perceive relevant changes or perturbations and to dynamically adapt biological functions accordingly (Gems & Partridge, 2008; Haigis & Yankner, 2010; Kenyon, 2005; Michaud et al., 2013). Adaptation to environmental and internal changes relies on molecular or cellular sensors, signal transduction pathways, and effectors that can dynamically modulate physiological functions (Kultz, 2005). While many signal transduction and response pathways that maintain the dynamic equilibria underlying biological robustness are well described, it is less clear why and how they decline in their effectiveness as organisms age. Even less is known about possible strategies to extend healthspan or lifespan by preserving the function of key components that become limiting for robustness.

We and others have observed that adaptive mechanisms that rely on changes in gene expression can become ineffective with age. Genes that are strongly responsive to external signals (e.g., oxidative stress, heat shock, pathogen epitopes) early in life, frequently lose that responsiveness in old animals (Ben‐Zvi et al., 2009; Bruunsgaard et al., 2001; Labbadia & Morimoto, 2015; Rahman et al., 2013). We noticed this phenomenon in studies on oxidative stress responses in Drosophila that are mediated by the transcription factor CncC, the fly homolog of mammalian Nrf2 (Rahman et al., 2013).

Nrf‐family transcription factors, including CncC, respond to cell stresses, such as increased ROS levels, xenobiotics, and unfolded proteins, by activating protective transcription programs to neutralize chemical threats and repair macromolecular damage. These protective and restorative functions preserve cell and tissue integrity and presumably underlie the anti‐aging functions of Nrf‐2 (Chatterjee et al., 2016; Motohashi et al., 2002; Motohashi & Yamamoto, 2004; Pitoniak & Bohmann, 2015; Sykiotis & Bohmann, 2008). Decreased Nrf2 function shortens lifespan in model organisms (Yoh et al., 2001) whereas genetic or pharmacological activation of Nrf2 can promote longevity, as demonstrated in experiments with Drosophila and *C. elegans* (Castillo‐Quan et al., 2016; Spiers et al., 2019; Sykiotis & Bohmann, 2008; Tullet et al., 2008). Thus, Nrf2 is considered an essential contributor to healthy aging and longevity.

Nrf2 and small Maf proteins form heterodimers, which bind to antioxidant response elements (AREs) in the promoters of cytoprotective genes (Moi et al., 1994; Motohashi et al., 2002). We have previously shown that Maf‐S, the only fly homolog of small Maf proteins, is required for the transcription activation function of CncC in Drosophila (Rahman et al., 2013). While Maf‐S resides constitutively in the nucleus and stays bound to AREs (Motohashi et al., 2004; Rahman et al., 2013), CncC only enters the nucleus after cells have been exposed to stress or other signals.

We found that the ability to stimulate the transcription of antioxidant and detoxification genes in response to CncC activation gradually diminishes and ultimately ceases in aging flies (Rahman et al., 2013). Interestingly, this loss of gene responsiveness can be ameliorated by boosting Maf‐S expression. Moderately increasing the abundance of Maf‐S throughout Drosophila development and adult life, by ubiquitous overexpression from a transgene, preserved the signal‐responsive expression of CncC target genes. Strikingly, this effect correlated with a delayed loss of fitness in old flies as assessed by several parameters, including stress tolerance, climbing ability and heart performance. These findings are consistent with the notion that the fitness of young organisms requires a robust system of dynamic adaptive processes that rely, at least in part, on the ability to activate genes in response to relevant signals.

Here we report experiments to explore the mechanisms underlying the decline of CncC‐mediated transcriptional stress responses in old adults and strategies to prevent this degenerative effect. We show that age‐associated changes of chromatin accessibility at AREs correlate with the loss of Nrf2 pathway responsiveness. Furthermore, overexpressing Maf‐S can delay the onset of such changes, which explains the preservation of the Nrf2 response in flies overexpressing Maf‐S. Similar effects were achieved by long‐term dietary supplementation with Oltipraz, a well‐tolerated Nrf2‐activating drug. Oltipraz treatment prevented the age‐associated loss of histone 3, lysine 9 acetylation in the promoters of Nrf2 target genes and preserved their transcriptional inducibility in response to stress signaling. These findings support the notion that hormetic mechanisms that serve to maintain the function and accessible chromatin structure of signal responsive genes can evoke long lasting, anti‐aging effects.

## 2 RESULTS

### 2.1 Irreversible loss of stress‐responsive gene expression in old Drosophila adults

We have previously shown that mild ubiquitous overexpression of the obligatory CncC dimerization partner Maf‐S can have beneficial effects on aging in Drosophila. Increased Maf‐S levels slow down the loss of stress‐responsive CncC target gene expression (Rahman et al., 2013) normally observed in older flies. Maf‐S overexpression did not have a notable effect on the basal or stress‐induced activity of these genes in young animals, but preserved their transcriptional competence and signal responsiveness in older adults.

**FIGURE 1:**
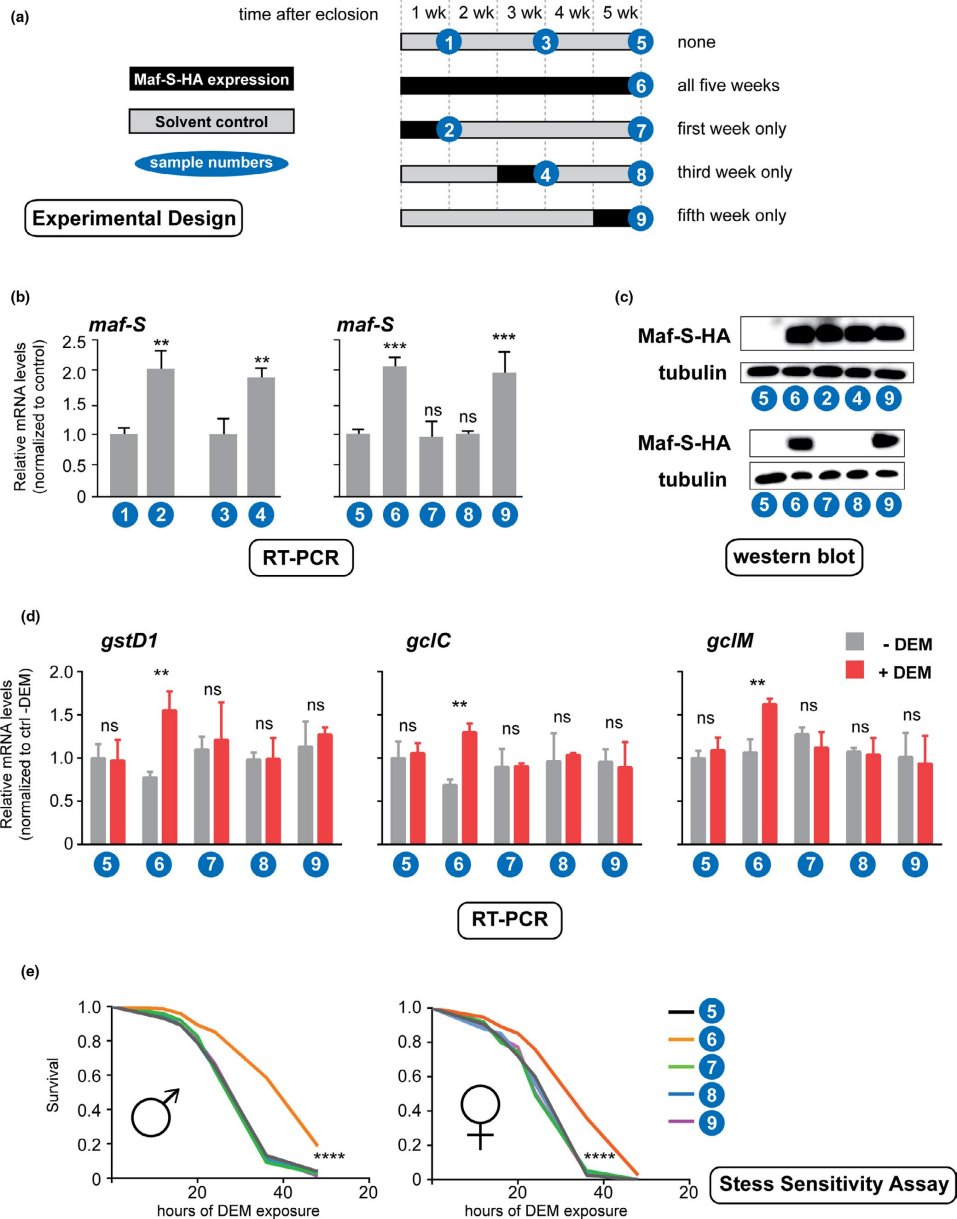
Irreversible loss of stress‐responsive CncC target gene expression in aging flies. (a) Experimental timeline. Transgenic Maf‐S expression was induced by RU486‐treatment of flies carrying the ubiquitously active inducible Gal4 driver Actin GeneSwitch‐Gal4 and a UAS‐driven Maf‐S expression construct (genotype Act‐GS‐Gal4, UAS‐Maf‐S‐HA). Control adult flies were maintained on standard food with the solvent alone (grey). Maf‐S overexpression was achieved by exposing flies to RU486‐containing food (black) for 5 weeks or for 1 week during the first, third, and fifth weeks. Flies were collected at different timepoints (marked with sample no.) for the experiments presented in panels b‐e. (b) RT‐qPCR experiments were performed to measure the expression of *maf*‐*S* mRNA at the indicated timepoints. *maf*‐*S* overexpression could be detected after RU486 exposure during week 1, 3, or 5 (compare samples 1, 3, and 5 with samples 2, 4, and 9, respectively). RT‐qPCR was performed to measure combined transcript abundance of endogenous and transgenic Maf‐S. Results were normalized to *actin5c* transcript levels. Fold activation relative to the mRNA levels in control flies is shown. ***, *p* < 0.001; ** *p* < 0.01 by 1‐way ANOVA followed by a multiple comparison test. ns, not significant. (c) Western blot experiments show the protein expression of transgenic Maf‐S (HA‐tagged) at the indicated timepoints. α‐tubulin was used as a loading control. (d) RT‐qPCR to quantify transcripts from CncC target genes (*gstD1*, Glutathione‐S‐transferase D1; *gclC*, Glutamate‐cysteine ligase catalytic subunit; *gclM*, Glutamate‐cysteine ligase regulatory subunit) under unstressed and 5 μM DEM‐induced stress conditions. ***, *p* < 0.001; ** *p* < 0.01 by 2‐way ANOVA followed by a multiple comparison test. ns, not significant. (e) Survival of male and female fly cohorts from treatment groups 5 to 9 (see panel a) after exposure to 5 μM DEM was recorded, and the data were analyzed by Mantel‐Cox log‐rank test. ****, *p* < 0.0001.

In the previous experiments, Maf‐S was continuously overexpressed under the control of the constitutively active armadillo‐Gal4 driver. We wanted to test whether such persistent over‐expression is required for maintaining an effective CncC response to stress and to confer fitness benefits to old flies. In other words, do elevated Maf‐S levels prevent or delay an irreversible loss of transcriptional competence, or do older flies simply need higher levels of Maf‐S to activate CncC driven gene expression programs in response to stress? To address these questions, we conducted experiments in which overexpression of Maf‐S was temporally controlled by the actin‐geneSwitch‐Gal4 (act‐GS‐Gal4) system, which can be activated by adding the steroid RU486 to the fly food (Osterwalder et al., 2001) (Figure 1a‐c). When such flies (genotype act‐GS‐Gal4; UAS Maf‐S) were reared in the absence of RU486, that is, without inducing Maf‐S overexpression, they lost the response of CncC target gene expression to acute oxidative stress, through exposure to dietary diethyl maleate (DEM), within 5 weeks of adult life (Figure 1d). This recapitulates the effect that was previously observed in wild type flies (Rahman et al., 2013). Next, we tested if persistent over‐expression of Maf‐S using the inducible system would reproduce the results of constitutive over‐expression and preserve stress responsive CncC target gene expression. Transgenic expression of Maf‐S was induced by oral administration of RU486 one day after hatching, and maintained until the animals reached the age of 5 weeks. At that time a brief exposure to DEM caused a robust activation of several direct CncC target genes, as measured by RT‐qPCR (Figure 1d). This result is consistent with our previously published findings that Maf‐S expression throughout life can preserve CncC target transcriptional competence. The outcome was different, however, when the flies were cultured on RU486 to induce Maf‐S overexpression for only one week. When transgenic Maf‐S expression was induced in the first, the third or the fifth week of life, a 2‐fold increase of Maf‐S levels was achieved similar to the overexpression observed after 5 weeks of continuous expression starting at day 1 after hatching (Figure 1b, c). Strikingly, however, this short‐term overexpression, regardless of the week of life in which it occurred, did not rescue the ability of old flies to activate CncC target gene expression in response to DEM treatment (Figure 1d). Maf‐S overexpression also had no effect on the already robust response of Nrf2 target genes to DEM exposure in young flies (Figure S1). We conclude that Maf‐S needs to be permanently overexpressed to counteract an irreversible age‐associated loss of transcriptional competence in CncC target genes. Taken together with the observation that endogenous Maf‐S mRNA levels do not appreciably change with age (Rahman et al., 2013), this result also indicates that the effects of Maf‐S overexpression are not simply explained by a restoration of Maf‐S protein levels that might have decreased below a healthy threshold in the aged organism or some of its organs.

To test whether continued Maf‐S overexpression also preserves a functional and dynamic chromatin structure at CncC responsive genes, we conducted ChIP experiments to monitor the histone H3 lysine 9 acetylation status of nucleosomes in the vicinity of ARE elements. The H3K9ac modification is a feature of transcriptionally active genes. As shown in Figure S2 (left panel), H3K9ac levels increase in the promoter regions of young flies after stress exposure. In contrast, 5‐week‐old control flies do not display this response. Consistent with the mRNA data (Figure 1d), long‐term Maf‐S overexpression preserves the stress‐responsive H3 K9 acetylation in CncC target gene promoters, but not in unrelated control regions. These results suggest that Maf‐S overexpression can preserve a functional epigenetic state at Nrf‐2 target promoters in aged flies, thereby facilitating efficient transcriptional activation in response to stress signals.

We tested if preservation of transcriptional competence of CncC target genes in old flies by continued Maf‐S overexpression correlated with increased fitness as assessed by their ability to withstand toxic levels of DEM. The animals in which Maf‐S had been overexpressed throughout life displayed marked resistance to DEM, whereas the expression of Maf‐S for 7 days in either week 1, 3, or 5 failed to confer this beneficial effect (Figure 1e).

One possible explanation for the failure of short‐term Maf‐S overexpression to rescue the functionality of CncC target loci in old flies would be its ineffective nuclear translocation. To test this possibility, we monitored the localization of Maf‐S‐HA that had been expressed throughout life or only for the last week of life. In both cases we observed indistinguishable levels of nuclear localized Maf‐S in several tissues tested (Figure S3). This result suggests that age does not impede the nuclear translocation of Maf‐S.

### 2.2 ARE accessibility declines with increasing age

**FIGURE 2:**
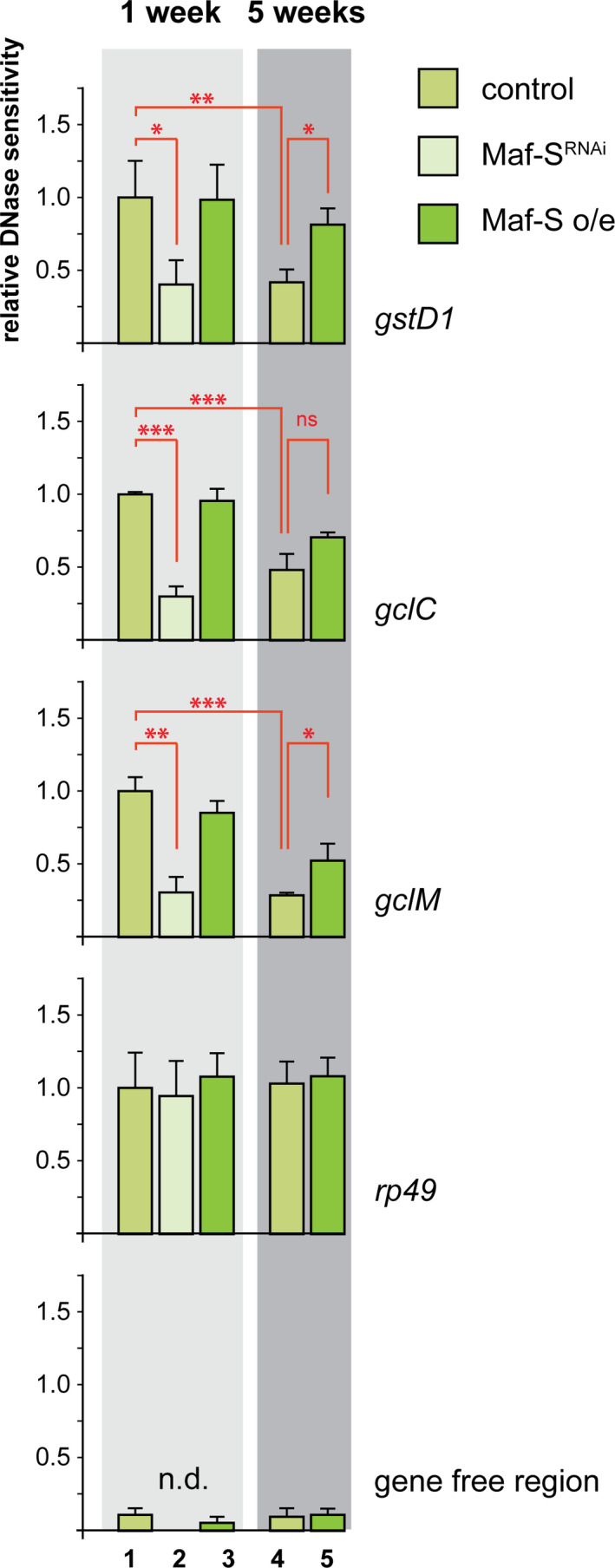
Maf‐S activity and age influence ARE accessibility at Nrf2 target promoters. Chromatin structure around the location of AREs in three CncC target gene promoters (*gstD1*, *gclC*, and *gclM*) was assessed by DNaseI‐qPCR assays (Follows et al., 2007). Young (lanes 1–3) and old flies (lanes 4 and 5) with wild‐type levels of Maf‐S expression (control, lanes 1 and 4), or in which Maf‐S was suppressed by ubiquitous expression of Maf‐S^RNAi^ (Maf‐S^RNAi^, lane 2), or in which Maf‐S was over expressed (Maf‐S o/e, lanes 3 and 5) were analyzed. All DNaseI hypersensitivity values for a given ARE expressed relative to the young controls, which were set to 1. Genotypes: control: arm‐Gal4; Maf‐S^RNAi^: arm‐Gal4, UAS‐Maf‐S^RNAi^; Maf‐S o/e: arm‐Gal4, UAS‐Maf‐S. A housekeeping gene *rp49*, and a gene‐free region were included as reference. *, *p* < 0.05; **, *p* < 0.01; ***, *p* < 0.001 by 1‐way ANOVA followed by a multiple comparison test. n.d. = not done

What could cause the irreversible age‐associated loss of transcriptional competence at Nrf2 target loci, and why would increased concentrations of Maf‐S delay this effect? Maf‐S is a constitutively nuclear protein that binds to ARE elements even in the absence Nrf2 activation when linked target genes are expressed at low basal levels (Blank, 2008; Rahman et al., 2013). We speculated that the Maf‐S protein serves as a “genomic placeholder” to maintain ARE‐containing genes in a transcriptionally competent state receptive for signal‐dependent activation by CncC. Maf‐S binding might keep these genes in a chromatin conformation that is permissive for CncC binding, H3K9 acetylation and engagement of the transcriptional machinery. We tested this prediction by conducting DNase I hypersensitive site mapping experiments to assess the accessibility of upstream regulatory regions of representative CncC target genes. These experiments were performed either in young (1 week old) or old flies (5 weeks old) in which Maf‐S activity had either been suppressed by expression of a dsRNA construct, or increased by constitutive transgenic over‐expression as previously described (Rahman et al., 2013). Whole flies were collected and processed for DNase I hypersensitive site analysis. Using qPCR, the DNase I accessibility of the transcription control regions surrounding the ARE elements of CncC target genes, as well as control genes, were analyzed (Figure 2). The results from these experiments support a number of conclusions:

#### 2.2.1 Chromatin accessibility at AREs is reduced in old flies

In young flies, CncC target gene promoters containing ARE elements are present in an open chromatin conformation typical of active and potentially active transcription control regions (Bell et al., 2011) (Figure 2, compare lanes 1 and 4). Comparison of the DNase I hypersensitivity around several CncC promoters in young and old flies shows that this open conformation is lost with increasing age. This observation suggests an erosion of chromatin organization at these loci, which coincides, and might be causal for, the loss of transcriptional competence of the CncC‐regulated genes in older adults, as shown in the previous experiment and in (Rahman et al., 2013). A constitutively expressed housekeeping gene, rp49, did not show this decrease in chromatin accessibility.

#### 2.2.2 Maf‐S function is required to maintain accessibility of ARE elements

RNAi‐mediated knock down of Maf‐S in young flies reduces DNase I hypersensitivity at ARE elements (Figure 2, compare lanes 1 and 2). This result supports the interpretation that Maf‐S is required to organize CncC target gene promoters in a chromatin state that permits access to DNase, and presumable to Nrf2 transcription factors.

#### 2.2.3 Maf‐S overexpression delays the loss of an open chromatin structure at CncC target gene promoters in aging flies

As reported above, flies in which Maf‐S is constitutively overexpressed under the control of a moderately active driver retain the ability to activate CncC target genes in response to stress at an old age, when wild type flies have lost this responsiveness. Consistently, we find that long term Maf‐S over‐expression preserves open chromatin confirmation at CncC target gene promoters as indicated by DNase I hypersensitivity (Figure 2, compare lanes 4 and 5). This finding suggests that the beneficial effect of elevated Maf‐S levels on stress‐responsive gene expression is mediated by a delay of age‐associated epigenetic decline. The accessibility of a housekeeping gene without apparent ARE elements, rp49, was not affected by Maf‐S levels, supporting the idea that the preservation of a transcriptionally competent epigenetic state at CncC binding sites is a direct effect of Maf‐S binding to AREs.

Based on the DNase I hypersensitivity experiments described above, we speculated that the chromatin state of ARE‐containing promoters changes with age so that they lose the ability to serve as binding sites for Nrf2 proteins, rendering associated genes unresponsive to CncC‐activating signals. We experimentally tested this interpretation using ChIP experiments in which we tested binding of Maf‐S and CncC to genomic ARE elements.

**FIGURE 3:**
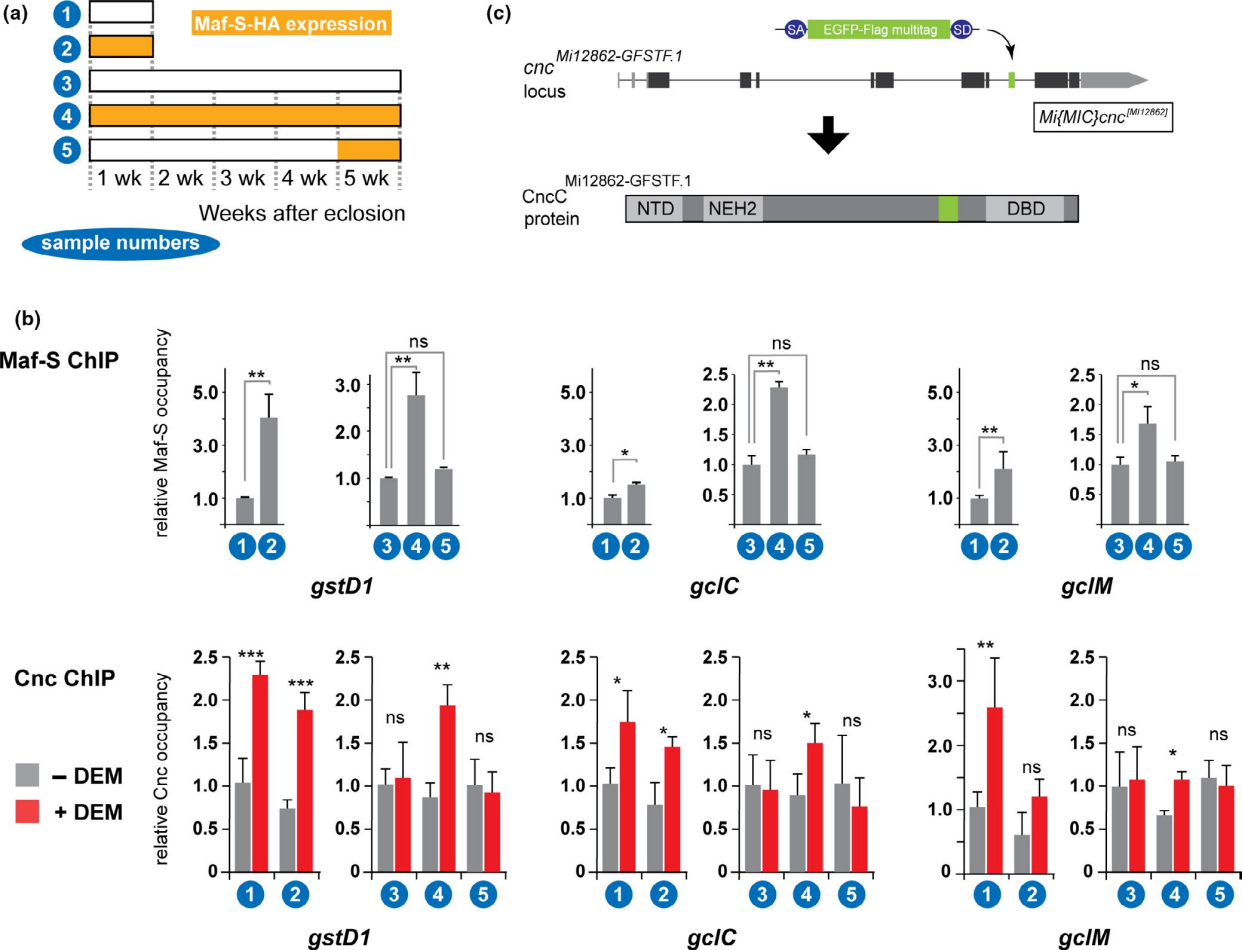
Loss of CncC and Maf‐S binding to chromatin in old animal can be precluded by long‐term Maf‐S expression. (a) Experimental timelines. *cnc^MI12862^* ^−^ *^GFSTF^* ^.^ *^1^* flies were crossed with act‐GS‐Gal4, UAS‐Maf‐S flies and the offspring (act‐GS‐Gal4, UAS‐Maf‐S; cnc^MI12862−GFSTF.1^) were maintained on solvent control or RU486‐containing food for one (young, #1 and #2) or five (old #3, #4 and #5) weeks before the ChIP experiment presented in b. The experiment in which Maf‐S binding was assessed was done in a stock carrying a wild‐type *cnc* allele. (b) HA‐tagged Maf‐S and flag‐tagged CncC binding at Nrf2 target promoters in young and old flies. Maf‐S and CncC enrichment at the promoters of three Nrf2 target genes *gstD1*, *gclC*, and *gclM* was assessed by ChIP‐qPCR. In the CncC ChIP experiment, 5 μM DEM was used to induce the promoter binding of CncC. Relative promoter occupancy was calculated by normalizing to the control group. *, *p* < 0.05; **, *p* < 0.01; ***, *p* < 0.0001 by 1‐way ANOVA (Maf‐S ChIP) or 2‐way ANOVA (Cnc ChIP) followed by a multiple comparison test. ns, not significant. (c) *cnc^MI12862^* ^−^ *^GFSTF^* ^.^ *^1^* flies carry a MIMIC element integrated in the *cnc* locus, which adds a new exon and results in an internal fusion of a EGFP‐Flag multitag to endogenous *cnc* gene products. The insertion site is depicted in the cartoon. SA, splicing acceptor; SD, splicing donor; NTD, N‐terminal domain; NEH2, Nrf2‐ECH homology 2 domain; DBD, DNA binding domain.

Five days after inducing the transgenic expression of epitope tagged Maf‐S in young animals (Figure 3a), the protein could be detected bound to genomic ARE elements (Figure 3b). Significant ARE‐occupancy could be observed in 5‐week‐old flies that overexpressed Maf‐S throughout adult life. However, when expression of the protein was induced in old flies for only the last 5 days of a 5‐week lifespan, no binding over background was detectable. This is not caused by a difference in transgene expression as the levels of transgenic Maf‐S in five‐week‐old adults after transient or chronic expression were comparable (see Figure 1b). This experiment shows that the effect of long‐term Maf‐S overexpression to maintain transcriptional competence of CncC target genes correlates with the preserved accessibility of the relevant ARE elements. In other words, Maf‐S overexpression is not sufficient to restore ARE access, once lost, in old flies.

Next, we wanted to test whether long‐term overexpression of Maf‐S would also affect stress‐inducible ARE binding of the endogenous CncC protein in old flies. For these experiments we employed a derivative of the Drosophila MIMIC line 12862. This strain carries an allele of *cnc*, *cnc^MI12862^* ^−^ *^GFSTF^* ^.^ *^1^*which is internally tagged with a MIMIC) (Nagarkar‐Jaiswal et al., 2015; Venken et al., 2011). The integration of the MIMIC element in the *cnc* locus adds a new exon that results in an internal fusion of an EGFP‐Flag multitag to the CncC gene product. Flies that are homozygous for the *cnc^MI12862^* ^−^ *^GFSTF^* ^.^ *^1^* allele are viable without any noticeable mutant phenotypes.

The *cnc^MI12862^* ^−^ *^GFSTF^* ^.^ *^1^* strain can be used to monitor interactions of CncC with chromosomal ARE sites at physiological levels of expression. To that end we conducted ChIP experiments using anti‐Flag antibodies in which we assessed the effect or oxidative stress, age and Maf‐S overexpression on CncC‐ARE interactions in vivo (Figure 3b). These experiments showed that CncC binding to the promoter regions of three target genes, *gstD1*, *gclC and gclM*, increases in young, 1‐week‐old, flies in response to oxidative stress (16 hrs oral exposure to DEM). Old flies at 5 weeks of age do not show this DEM response; in these aged animals CncC binding is not inducible by stress. However, 5‐week‐old flies in which Maf‐S has been overexpressed throughout the aging process, retain significant DEM‐responsive CncC binding to AREs. Consistent with the functional data shown above, overexpression for just the last week of life is not sufficient to rescue the stress‐inducible ARE binding in old animals. This interpretation is consistent with the age‐associated and irreversible loss of chromatin accessibility and DNase I hypersensitive sites at genomic loci where AREs reside.

### 2.3 Pharmacological preservation of the Nrf2 response in aging flies

The experiments described above indicate that, at least in principle, it is possible to stall epigenetic decline at CncC target genes and the associated loss of their stress responsiveness by overexpressing Maf‐S. As shown before (Rahman et al., 2013), such a manipulation can counteract age associated decline of fitness. However, transgenic Maf‐S overexpression would not be very translatable to a therapeutic setting. Therefore, we explored the feasibility of achieving similar effects through a pharmacological approach. Could active transcription maintain functional chromatin with age? To test this idea, we activated the transcription of CncC target genes by oral administration of the organosulfur compound Oltipraz, which effectively increases Nrf2 target gene expression, but does not cause cell stress. It is for this reason that Oltipraz and similarly acting compounds can serve as cancer‐chemopreventive or anti‐inflammatory drugs (Chatterjee & Bohmann, 2012; Chatterjee et al., 2016; Iida et al., 2004; Kensler et al., 1999; Sykiotis & Bohmann, 2008). We have shown that oral dosing with Oltipraz can activate CncC in Drosophila (Sykiotis & Bohmann, 2008) and that administration of the drug is well tolerated in adult flies, even for extended periods of time. Importantly, long‐term treatment of adult flies with Oltipraz has no discernable effect on food intake, fertility or fecundity (Figure S4).

**FIGURE 4:**
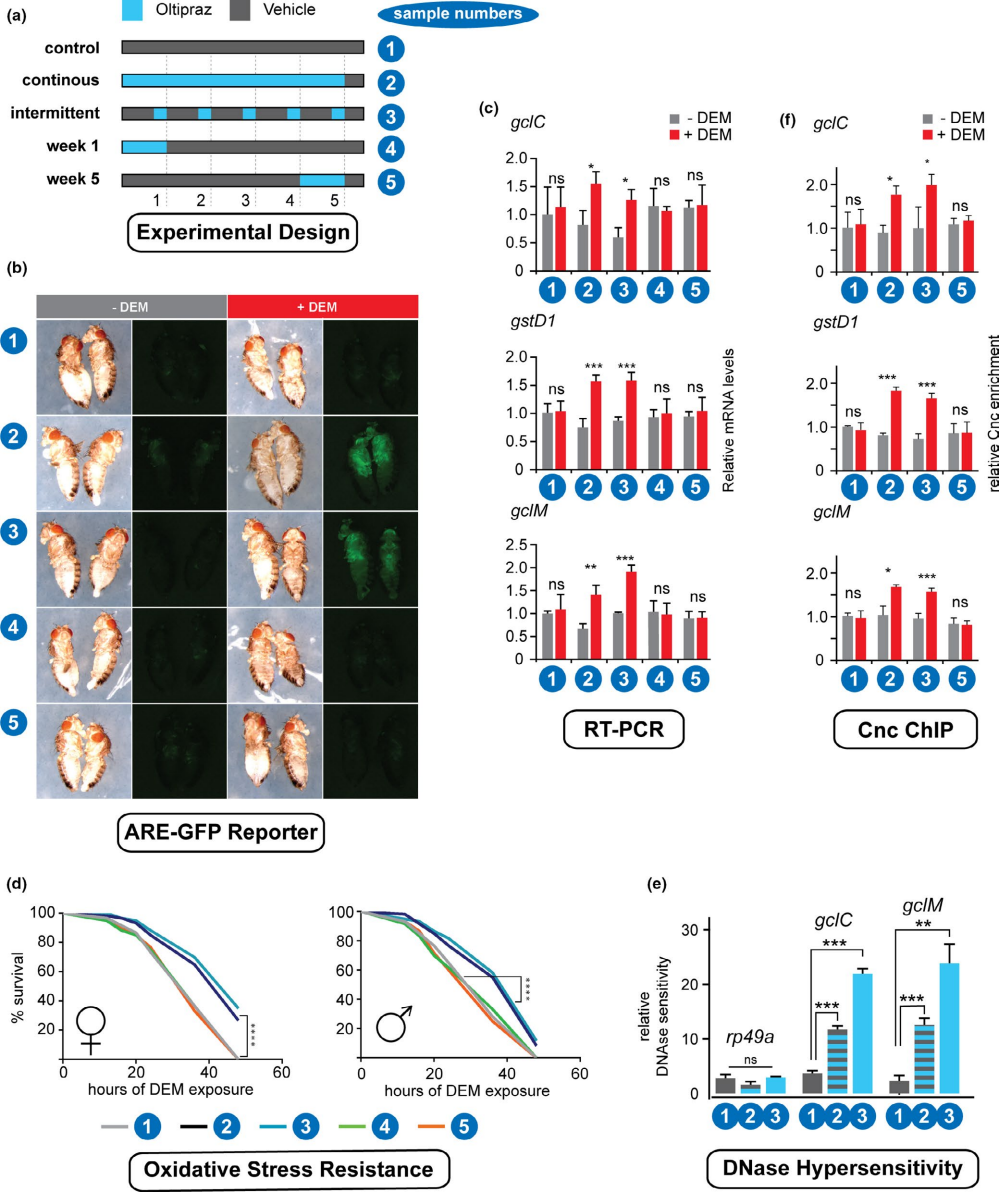
Continuous or intermittent Oltipraz exposure during adult life preserves Nrf2 target gene signal responsiveness and chromatin structure. (a)Experimental timeline. Control flies were kept on food without Oltipraz (#1). Long‐term oltipraz treatment includes continuous (#2) and intermittent (#3) strategies. Another two groups of flies were exposed to 1‐week Oltipraz treatment during week 1 (#4) or week 5 (#5). Note that by the end of week 5 all flies were transferred to normal food for 3 days before being subjected to the later experiments. (b)CncC reporter activity in Oltipraz the five groups of flies. Flies carrying the CncC responsive ARE: GFP reporter (Chatterjee & Bohmann, 2012) were subjected to different Oltipraz treatment regimens described in panel a. ARE: GFP reporter activity were assessed under basal and stressed (5 μM oral DEM) conditions. (c) RT‐qPCR quantifying CncC‐regulated mRNAs *gstD1*, *gclC*, and *gclM* in the five groups of flies described in panel a. ***, *p* < 0.001, **, *p* < 0.01; *, *p* < 0.05 by 2‐way ANOVA followed by a multiple comparison test. ns, not significant. (d) Survival of the five experimental groups after exposure to 5 μM DEM was recorded, and the data were analyzed by Mantel‐Cox log‐rank test. ****, *p* < 0.0001. (e) Chromatin organization at promoter regions of two CncC target genes *gclC*, *gclM* and a housekeeping gene *rp49* was assessed by DNase I ‐qPCR assay. * = *p* < 0.05; **, *p* < 0.01, ***, *p* < 0.001 by 1‐way ANOVA followed by a multiple comparison test. ns, not significant. (f) Flies carrying the *cnc^MI12862^* ^−^ *^GFSTF^* ^.^ *^1^* MIMIC element which express epitope tagged CncC from the endogenous promoter, as described in Figure 3, where exposed to control food or treated continuously, intermittently or only during week 5 with oltipraz (see panel a). Subsequently, DEM treatment and ChIP‐PCR was conducted as described for the experiment in Figure 3. Note that DEM inducible CncC binding is observed in 5‐week old flies that had continuously and intermittently received Oltipraz, but not in the controls. ***, *p* < 0.001, **, *p* < 0.01; *, *p* < 0.05 by 2‐way ANOVA followed by a multiple comparison test. ns, not significant.

For the experiment shown in Figure 4, adult flies were exposed to different Oltipraz treatment regimens (schematically described in Figure 4a). One group was maintained on food supplemented with 500 µM Oltipraz throughout the experimental 5‐week lifespan (continuous). A second group was transferred from normal food to Oltipraz food for three days every week (intermittent). The final two groups were exposed to Oltipraz for just the first or the last week of life. Flies that were not treated with Oltipraz were kept on food containing a corresponding amount of the solvent as control. The different treatments were maintained until the flies reached 5 weeks of age. At that stage they were switched to standard food for three days after which CncC activity, as assessed by ARE reporter readout or by quantifying target gene expression, was detected at a similar basal level in all treatment groups. Subsequently, the different experimental cohorts were either exposed to oxidative stress (oral DEM) or mock‐treated. The animals were then analyzed for the following parameters:

1. Oxidative stress‐inducible CncC target gene expression. After being subjected to the various Oltipraz treatment courses, flies carrying an ARE‐GFP CncC activity reporter (Chatterjee & Bohmann, 2012) were exposed to acute oxidative stress (dietary DEM) or mock treated. Subsequently, the activity of the ARE reporter response was examined by observing the flies under UV illumination (Figure 4b). In parallel expression of prototypical CncC target genes was measured by RT‐PCR (Figure 4c). As in previous experiments, the transcriptional induction of the GFP reporter and of CncC target genes was negligible in the 5‐week old control flies. However, both the continuously and the intermittently treated cohorts of the same age showed robust up‐regulation of the reporter and of all the tested genes. Short‐term treatment, either in week 1 or in week 5 did not restore the DEM response in old flies.
2. Stress sensitivity. Flies from all treatment groups were exposed to lethal doses of DEM and the time of their survival was measured (Figure 4d). The control flies and flies treated with Oltipraz for only 1 week showed the high degree of stress sensitivity, typical for 5‐week old flies. However, the cohorts treated intermittently or continuously with Oltipraz were significantly more resistant to acute DEM stress and responded like younger flies.
3. DNase hypersensitivity (Figure 4e). The upstream region of CncC target genes showed more DNase I hypersensitivity in chromatin from old flies after intermittent or continuous Oltipraz exposure compared to mock treated control animals. This indicates an open chromatin conformation at CncC target genes in the Oltipraz treatment groups. Thus, Oltipraz exposure preserves chromatin conformation as it is normally found in younger flies. The DNase I hypersensitivity profile around the housekeeping gene, *rp49*, which is not regulated by CncC, was not affected by the treatments.
4. CncC ChIP (Figure 4f). Animals carrying the epitope‐tagged *cnc^MI12862^* ^−^ *^GFSTF^* ^.^ *^1^* allele introduced above were exposed to the intermittent, continuous, or delayed Oltipraz treatment (week 5 only) or mock treated as shown in Figure 4a. At 5 weeks of age, half of each treatment group was exposed to acute oxidative stress (DEM‐laced food for 16 hours) while the other half was mock treated. ChIP qPCR experiments were performed to monitor stress‐induced binding of CncC to the ARE elements of *gstD1*, *gclC* and *gclM*. Consistent with the data described above, no significant DEM‐induced binding of CncC to AREs was detectable in old control flies (Figure 4f, #1). However, such stress‐induced interactions were detected in flies of the same age that had been exposed to Oltipraz, either intermittently or permanently throughout adult life, but not after treatment for just the last week. We conclude that activation of the CncC signaling pathway throughout life can maintain genomic ARE elements in an accessible and functional epigenetic conformation such that organisms retain the ability to adapt to conditions of cell stress.
5. Epigenetic response to stress exposure (Figure S2, right panel). To test if Oltipraz treatment can preserve stress responsiveness also at the chromatin level, we assessed the H3K9ac state of CncC target gene promoters and unrelated genomic regions in aged flies exposed to different Oltipraz treatment schedules (Figure 4a). Figure S2 shows that also at the level of this transcription‐associated histone mark, continuous or intermittent Oltipraz treatment has effects that recapitulate those of long‐term Maf‐S overexpression. Stress‐dependent increases of H3K9ac at AREs are retained in old flies with continuous or intermittent Oltipraz, but not with the last‐week treatment. We conclude that the salutary effect of both treatments is related to maintaining a “youthful” chromatin state.

The experiments described above indicate that Oltipraz exposure, like Maf‐S overexpression needs to occur throughout life to exert its beneficial effects. To test this conclusion with more timepoints, we assessed the effect of staggering the onset of Oltipraz exposure in weekly increments (Figure S5). These experiments show that the later Oltipraz feeding commences, the less benefit is observed in old flies. We conclude that Oltipraz acts similar to Maf‐S over‐expression by delaying a progressive decline in transcriptional competence, but that it cannot reverse such a loss of Nrf2 target gene responsiveness.

### 2.4 Oltipraz treatment can extend lifespan

**FIGURE 5:**
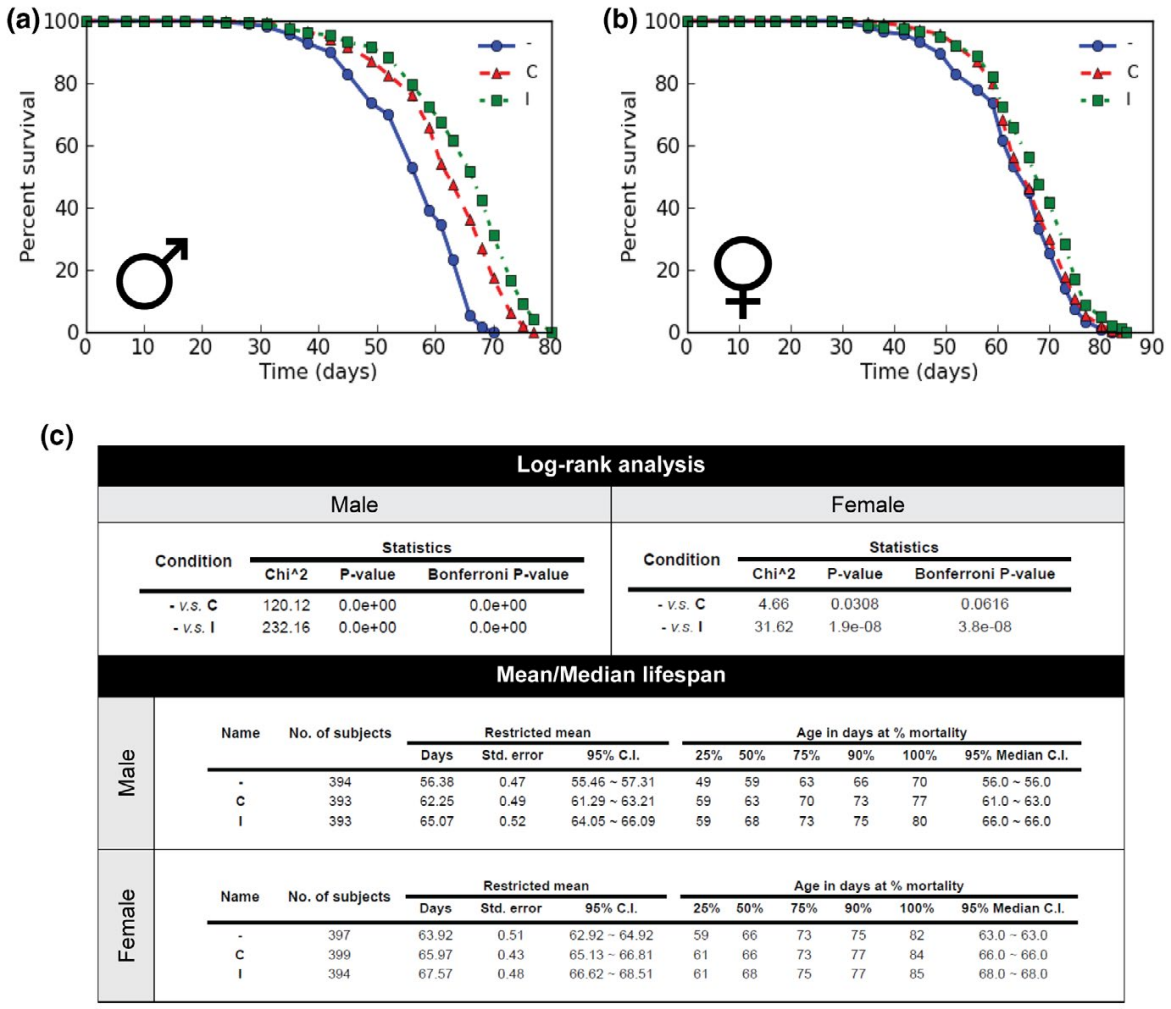
Oltipraz treatment extends lifespan. W1118 flies were collected and subjected to the indicated Oltipraz treatment regimens. A total of 400 flies (males and females) from each condition were monitored for lifespan. Results were displayed as survival proportion and were analyzed by Mantel‐Cox log‐rank test. *, *p* < 0.05; ****, *p* < 0.0001. ‐, control treatment; C: continuous treatment; I, intermittent treatment. The experiment shown here was repeated twice with reproducible results. (a) Survival curve of male files. (b) Survival curve of female files. (c) Statistical analysis of the lifespan assay. Log‐rank tests were performed on the survivorship data using OASIS2 software.

The preservation of CncC target gene responses in old flies after continuous or intermittent Oltipraz exposure and their increased fitness, as indicated by oxidative stress tolerance, suggested that this treatment might slow down aging. To directly test this idea, we conducted lifespan experiments under the different Oltipraz treatment protocols used in the experiments described above. Separate cohorts of male and female w1118 flies were kept on control food or exposed to the continuous or intermittent Oltipraz feeding protocol. As shown in Figure 5a, male w1118 flies exposed to intermittent Oltipraz treatment lived on average 10 days longer than controls, corresponding to a 14.3% extension of lifespan with a p‐value below 0.0001 by log‐rank test. Continuous feeding reproducibly resulted in a somewhat less effective, but still significant lifespan extension (7 days, 10%, *p* < 0.0001 by log‐rank) (Figure 5c). While the effects were less pronounced, females also showed a trend toward increased lifespan, which did reach statistical significance in the intermittent feeding condition (3.7 days, *p* < 0.001) (Figure 5b and c). In conjunction with the previous experiments, these data demonstrate that periodic treatment with Nrf2 activating drugs can prolong both healthspan, that is, improve fitness of older individuals, and lifespan.

## 3 DISCUSSION

Epigenetic information is encoded in the complex chromatin organization formed by different functional domains, areas of silenced genetic material, enhancers, boundary elements, histone modifications, poised polymerase and other landmarks. A well‐organized epigenome therefore contains information that is critical for organismal function and homeostasis. Epigenetic aging is a collective term for different types of changes in this chromatin organization that cause a loss of this information over the course of an organism's lifetime. During replicative aging in budding yeast, for example, old mother cells suffer a general loss of nucleosomes (Feser et al., 2010; Hu et al., 2014). Depletion of heterochromatin markers occurs as flies and other organisms age (Tsurumi & Li, 2012). Several studies document how specific patterns of epigenetic histone marks become diffuse with increasing age (Lopez‐Otin et al., 2013). These epigenetic changes correlate with, and may be causal for, aging and associated functional decline. An erosion of chromatin organization can be expected to disrupt gene regulation, increase genomic instability and interfere with replication. It is important to understand the nature and effects of epigenetic aging and to explore mechanisms that might preserve epigenetic integrity.

Several studies point to a connection between defective Nrf2 function and epigenetic aging. Hutchinson‐Gilford Progeria Syndrome (HGPS) causes the accelerated manifestation of geriatric symptoms and premature death. It is caused by a mutant version of lamin A, referred to as progerin. Cells expressing progerin display irregular nuclear architecture and defects in chromatin structure. They are highly sensitive to oxidative stress and show differentiation defects (Zhang et al., 2011). The correlation of accelerated aging with the breakdown of chromosome organization has established HGPS as a model system for exploring the mechanisms and effects of epigenetic aging (Arancio et al., 2014). Kubben et al. showed that progerin interferes with Nrf2 function and that genetic or pharmacological activation of Nrf2 can reverse several of the cell‐aging phenotypes associated with HGPS (Kubben et al., 2016). These findings support a role of Nrf2 in counteracting epigenetic aging.

We show here that a “youthful” chromatin state of CncC target loci, apparent by their accessibility and their signal responsive H3 K9 acetylation, can be preserved in aging flies by the overexpression of Maf‐S, as well as by persistent or intermittent Oltipraz treatment during fly lifespan. As a result, transcriptional competence of CncC target genes and systemic response to oxidative damage are more efficient in these flies.

Maf‐S binds to AREs even in the absence of signaling and gene activation, and it is plausible that its function in this context is to maintain the region in an accessible, CncC‐responsive state. Increased abundance of Maf‐S might conceivably make this function more robust and less vulnerable to stochastic perturbations. It is interesting to note that the transcriptional program regulated by CncC leads to the expression of proteins that protect cells from oxidative stress and challenges to proteostasis. It has been shown that this function can counteract the disruption of functional chromatin structures caused by oxidative damage (Kreuz & Fischle, 2016). The preservation of CncC‐dependent gene expression as a result of Maf‐S overexpression or Oltipraz treatment may in this way have a broader effect on epigenetic aging and also preserve the organization of genes that are not directly targeted by CncC.

We surmise that the longevity promoting effect of exposing flies to dietary Oltipraz throughout life is causally related to the observed preservation of CncC’s ability to sense external signals and to activate transcription. This would facilitate continued dynamic anti‐oxidant, anti‐inflammatory and detoxifying responses in old age. It is interesting to note that the salutary effects of Oltipraz feeding on lifespan are more pronounced in males than in females. This sexually dimorphic response is consistent with similar observations in experiments in which the effect of genetically stimulated CncC activity on longevity and oxidative stress resistance were measured (Sykiotis & Bohmann, 2008). In these experiments too, males showed a more pronounced benefit of CncC gain of function. Different responses of male and female flies to various interventions intended to prolong lifespan have been reported before (Bauer et al., 2005; Wang et al., 2005). While the reason for this dimorphism is not clear, it may be related to the marked metabolic differences between males and the larger, egg‐producing females.

Another interesting observation is that intermittent Oltipraz feeding (3 days on/4 days off each week) is sufficient to extend lifespan. In fact, this intermittent regimen is slightly more effective than continuous dosing with the compound. It can be speculated that occasional activation of CncC responsive genes is sufficient to maintain them in a functional state, while the persistent exposure to Oltipraz may have a minor adverse effect on the animals. This might explain the somewhat stronger effect of the intermittent treatment.

Transcriptional memory is a phenomenon that explains how cell‐ or tissue‐specific gene expression programs can be maintained over long periods of time and multiple cell divisions, even after the cues that established this expression have ceased. A paradigmatic case for transcriptional memory is the maintenance of Hox gene expression domains by Polycomb and Trithorax proteins throughout Drosophila development (Francis & Kingston, 2001). This and other examples have highlighted the importance of epigenetic organization as a basis for transcriptional memory. Memory mechanisms can not only facilitate the long‐term expression of specific genes, but they may also maintain genes at low activity, but in a poised state ready for up‐regulation in response to appropriate signals. Our data suggest that such a poised state can be lost with age and that a resulting loss of transcriptional competence might contribute to age‐associated frailty. Our findings suggest, however, that the probability of such functional decline can be decreased by frequent gene activation. Several observations suggest that such a “use it or lose it” model might also operate in humans. In other words, periodic stimulation of the Nrf2 pathway might preserve its function in aging people and contribute to a longer health span. For example, exercise has been shown to activate Nrf2 (Done & Traustadottir, 2016; MacNeil et al., 2012). Interestingly, studies on older adults suggest that physically active individuals display more robust Nrf2 activation than age‐matched sedentary controls (Safdar et al., 2010). It would be compatible with our model that regular activation of Nrf2 by exercise would keep stress defense genes responsive. Comparing the epigenetic landscape of people that are regularly exercising to sedentary ones might be informative.

An important conclusion that can be drawn from our data is that aging can occur at the level of the gene, and that such epigenetic aging, manifested in the loss of transcriptional mechanisms of adaptability, could be a fundamental cause of age‐associated functional decline. The important question of what the mechanistic basis for this loss of genetic responsiveness and its preservation in the presence of overexpressed Maf‐S or Oltipraz exposure is remains to be addressed. Our findings show that ARE sites in the genes of older animals lose accessibility to CncC, even though the transcription factor is still expressed and can translocate to the nucleus. Possible causes for this “aging at the level of the gene” include changes in chromatin organization at the level of nucleosome positioning, or overall nuclear topology. The observation that the dynamic regulation of H3 K9 acetylation is lost with age raises the question if alterations of other, possibly non‐signal‐dependent, histone modifications or binding of non‐histone proteins are also affected. Systematic epigenetic mapping studies in young and old animals under different Oltipraz feeding conditions should be conducted to explore these important issues further.

Another question that should be addressed is whether the loss of Nrf2 target gene responsiveness with increasing age that we describe here for a number of archetypal oxidative stress response gene is shared with other inducible transcription units, whether they are regulated by Nrf factors (e.g., proteasome subunits, certain metabolic genes) or by other transcription factors (AP‐1, NFκB, p53 etc). It would also be of interest to determine if tissue differences exist in terms of epigenetic aging. For example, is this process more prevalent in soma versus germline, or in high turnover tissues (skin, immune cells) versus more quiescent tissues?

Importantly, the finding that the epigenetic decline that we describe here can be modulated by pharmacological or dietary interventions may provide new impulses for developing rationally designed anti‐aging strategies. In this regard, it is interesting to note that many phytochemicals that have reported health‐promoting effects including polyphenols, fish oil, isothiocyanates from broccoli, cabbage, and other cruciferous foods are known activators of Nrf2 (Pall & Levine, 2015). One might speculate that these compounds could have an effect similar to that of Oltipraz in our long‐term feeding experiments and can maintain the Nrf2 system in a functional state even as the organism ages.

## 4 EXPERIMENTAL PROCEDURES

### 4.1 Fly stocks and maintenance

arm‐Gal4, UAS‐Maf‐S and UAS‐Maf‐S^RNAi^ flies have been described in Rahman et al., 2013. The Actin GeneSwitch‐Gal4 stock is available from the Bloomington stock center. *cnc^MI12862^* ^−^ *^GFSTF^* ^.^ *^1^* flies were generated as follows: Vectors pBS‐KS‐attB1‐2‐PT‐SA‐SD‐[phase 1]‐EGFP‐FlAsH‐StrepII‐TEV‐3xFlag and ΦC31 integrase were injected into MI12862 embryos. The F0 flies were crossed to balancer virgins or males of y* w*; D/TM6b, Hu, Tb or FM7j, B[1]. Transgenic F1 flies were scored for the loss of yellow+ (yellow−phenotype) and crossed to balancer virgins of y* w*; D/TM3, Sb, Tb. Transgenic F2 flies were intercrossed to establish the final stock. Correct RMCE events were confirmed by PCR assay as described in Nagarkar‐Jaiswal et al., 2015.

In experiments using RU486 treatment, 10 mM RU486 (Cayman Chemical Company, 10006317) was prepared in 80% ethanol and mixed with standard fly food at 50°C. 100 µM RU486 containing food was used for long‐term treatment while 320 µM RU486 food was used for short‐term treatment. A corresponding amount of ethanol was added to the control food.

In experiments using Oltipraz treatment, 50 mM Oltipraz (LKT Laboratories, Inc., 20020822) was prepared in DMSO and mixed with standard fly food at 50°C to a final concentration of 500 µM. A corresponding amount of DMSO was added to control food. In all Oltipraz experiments, flies were transferred to standard (no Oltipraz) food 3 days prior to experiment to avoid high basal levels of Nrf2 activity.

During DEM treatments, we used 5 mM DEM solution in assays of old flies and 20 mM DEM for young flies, due to the age‐related difference in DEM response. DEM was dissolved in ethanol to a final concentration of 10% and then mixed with 5% sucrose. 20 – 25 flies were transferred into a vial containing a piece of filter paper soaked with DEM or solvent. Flies were kept on DEM for 14 to 16 hours and collected for mRNA or ChIP assays. Note that during the DEM treatment, flies were no longer exposed to RU486‐ or Oltipraz‐containing food.

### 4.2 Primer sequences

Primer pairs for amplification of *maf*‐*S*, *gclC*, *gstD1* transcripts were described in Rahman et al., 2013. Primers for *gclM* and *act5C* are listed below: *gclM*, 5’‐CGGTGCAAAGTGTATTTAAGTGG‐3’ and 5’‐TGGTCGGTATCATGGTGACA‐3’; *act5C*, 5’‐CCTGGCATCGCCGACCGTAT‐3’ and 5’‐AGTACTTGCGCTCTGGCGGG‐3’.

Primer pairs flanking the promoters of CncC targets are listed below: *gclC* ARE, 5’ GTTACCCACTGATAGAAGCCACAA‐3’ and 5’‐ ACAAAAGGAGATAGATTCGCACGA‐3’; *gclM* ARE, 5’‐CCCGTCCACACATCCTAGTT‐3’; and 5’‐CGGTTTCCAGTTGTAACTGTTG‐3’; *gstD1* ARE, 5’‐ AGGCAGCTCTTGTAATTTCTTGTT‐3’ and 5’‐ATATGTCGAGATTTGCTTCCTTTT‐3’. Primers for *rp49* promoter region are 5’‐GCGCCCAAGTTTAAATTCAT‐3’ and 5’‐ATTCGCCGATAGTTTCGTTC‐3’.

### 4.3 Chromatin immunoprecipitation (ChIP)

We followed the ChIP protocol described in (Alic et al., 2011) with some modifications. 200 female flies were collected, snap frozen in liquid nitrogen and ground to a fine powder using pestle and mortar. Formaldehyde was added to a final concentration of 0.5% for crosslinking, followed by 2.5 M glycine to stop the reaction. Samples were sonicated using a Covaris Sonicator S2. The majority of the resulting DNA fragments ranged in size between 200 bp and 500 bp as determined using agarose gel electrophoresis.

20% of the sonicated chromatin was saved as input material and stored at −80°C. The remainder of the samples were used for ChIP followed by steps described in Alic et al., 2011. Anti‐H3K9ac polyclonal antibody (Millipore), anti‐Flag monoclonal antibody (M2, Sigma‐Aldrich) and anti‐HA polyclonal antibody (Sigma‐Aldrich) were used in H3K9ac, Cnc and Maf‐S ChIP, respectively. Quantitative PCR was performed to detect the enrichment of specific DNA sequences.

### 4.4 DNase I hypersensitive site mapping

Nuclei from 200 adult flies were extracted (Wilson et al., 1995) and the resulting nuclear suspensions were separated into mock‐ or DNase I‐treated groups. 10 µL DNase I (2000U/mL) and 10 µL 10x DNase I buffer (New England BioLabs, M0303S and B0303S) were added to the DNase I‐treated nuclear suspension in a final volume of 100 µL, and only 10x DNase buffer was added to mock‐treated groups. Reactions were incubated in a 37°C water bath for 10 – 15 min. The subsequent DNA cleaning and library preparations steps are described in (Follows et al., 2007). DNA from mock‐treated samples was purified and used as control for final normalization analysis.

### 4.5 mRNA quantification and Stress Sensitivity assays

RNA extraction, cDNA preparation and quantitative PCR were performed as described in Sykiotis & Bohmann, 2008.

Oxidative stress resistance of adult flies of different genotypes was assessed as previously described (Sykiotis & Bohmann, 2008). Log‐rank tests were performed on the survivorship data using GraphPad Prism software.

### 4.6 Feeding and fertility assays

To assess the feeding behavior of flies, we collected 2‐week old flies and placed them on standard food ±blue food coloring for 16 hours. 10 flies from each treatment were squished in PBS +1% triton X‐100 buffer. The food intake was quantified by measuring absorbance at 630 nm using a Nanodrop spectrophotometer (Deshpande et al., 2014).

To assess the reproductive capacity of flies, we collected 2‐week old adults and placed them into fly cages over apple juice plates. Fecundity was determined by counting embryos per female present on the apple juice plates immediately after 12 h dark or 4 h daytime periods. Fertility was determined by calculating the ratio of hatched to unhatched embryos 2 days after egg‐laying.

### 4.7 Immunostaining and Microscopy

MafS‐HA flies of indicated age and exposure were collected and dissected in PBS. Fly tissues were fixed in PBS +4% formaldehyde followed by immunostaining procedures as described in Chatterjee et. al, 2016. Anti‐HA (3F10, monoclonal) was used at the concentration of 1:2000. Hoechst was used to stain DNA. Confocal images were collected using a Leica SP5 confocal system and processed using Image J and Adobe Photoshop.

### 4.8 Lifespan analysis

Flies were collected at day 0 to day 1 post‐eclosion. Males and females were kept together overnight to ensure the same mating status. They were then separated by sex and subjected to the desired experimental treatment. In each group, 400 flies distributed into 8 bottles of control food or food containing 500 μM Oltipraz were monitored over time. In the intermittent group, flies were exposed to Oltipraz food three days/week (Friday to Monday). Flies were kept at consistent temperature (24 ± 2 °C) and humidity (55%–70%) with a 12:12 hour light‐dark cycle and were transferred to fresh food three times per week. Flies were scored as live or dead twice per week till the last survivor was dead. Log‐rank tests were performed on the survivorship data using OASIS2 software (Han et al., 2016).

## CONFLICT OF INTEREST

The authors declare no conflict of interest.

## AUTHOR CONTRIBUTIONS

All authors contributed to conception and experimental design, data analysis, and interpretation. YC and AP conducted all experiments and helped with manuscript preparation. JW generated the MIMIC tagged Cnc line used for ChIP experiments. YC and DB co‐wrote the manuscript. All authors commented on the manuscript.

## Supporting information

FIGURE S1

FIGURE S2

FIGURE S3

FIGURE S4

FIGURE S5

## Data Availability

The data that support the findings of this study are available from the corresponding author upon reasonable request.
