# Supplementary figures and images for: Preserving transcriptional stress responses as an anti‐aging strategy
Source: Aging Cell. 2021 Jan 20;20(2):e13297. doi: 10.1111/acel.13297 (PMC7884037; doi:10.1111/acel.13297)

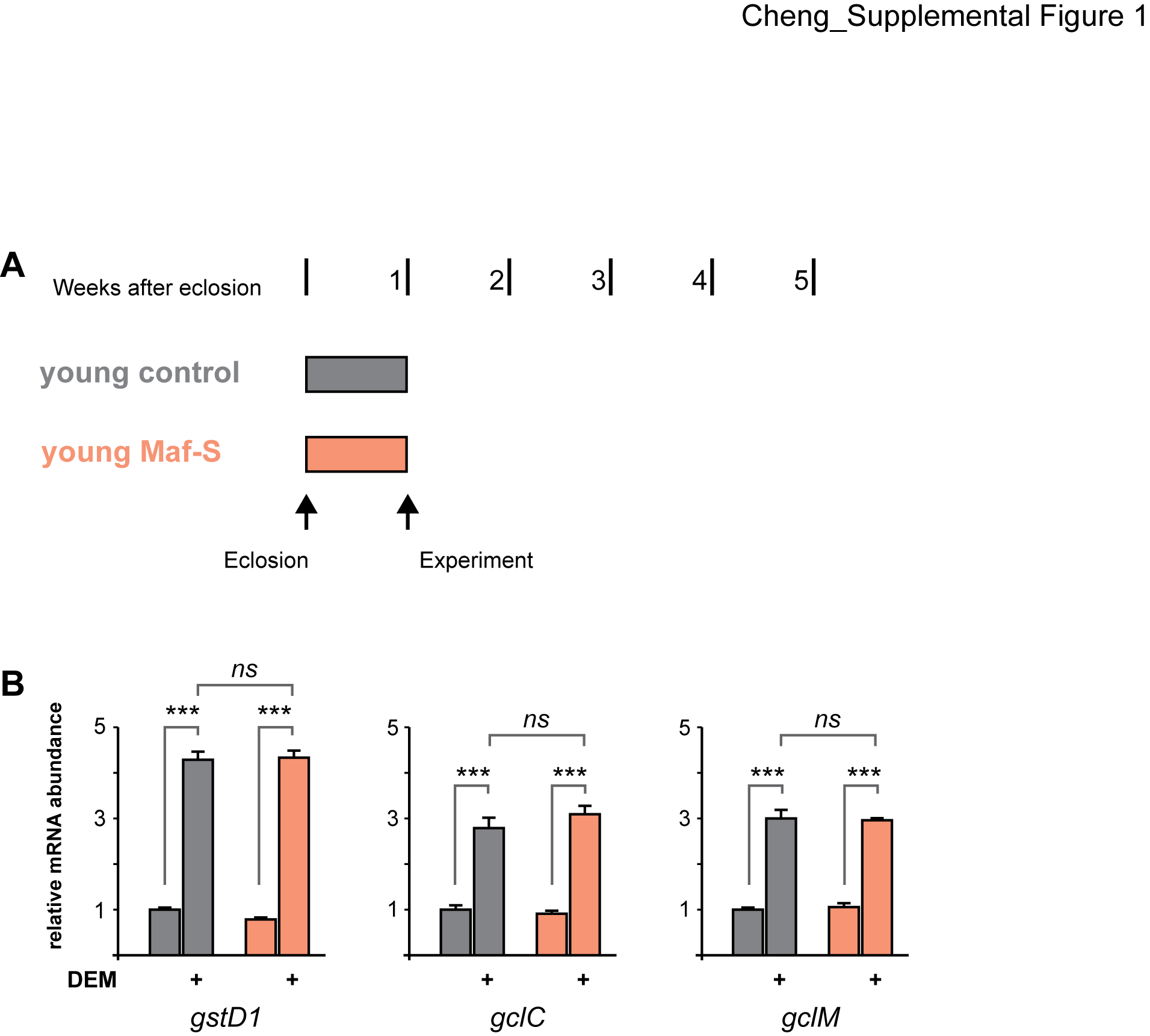

Supplement: Supplementary file 1 — FIGURE S1 [file ACEL-20-e13297-s001.tif]

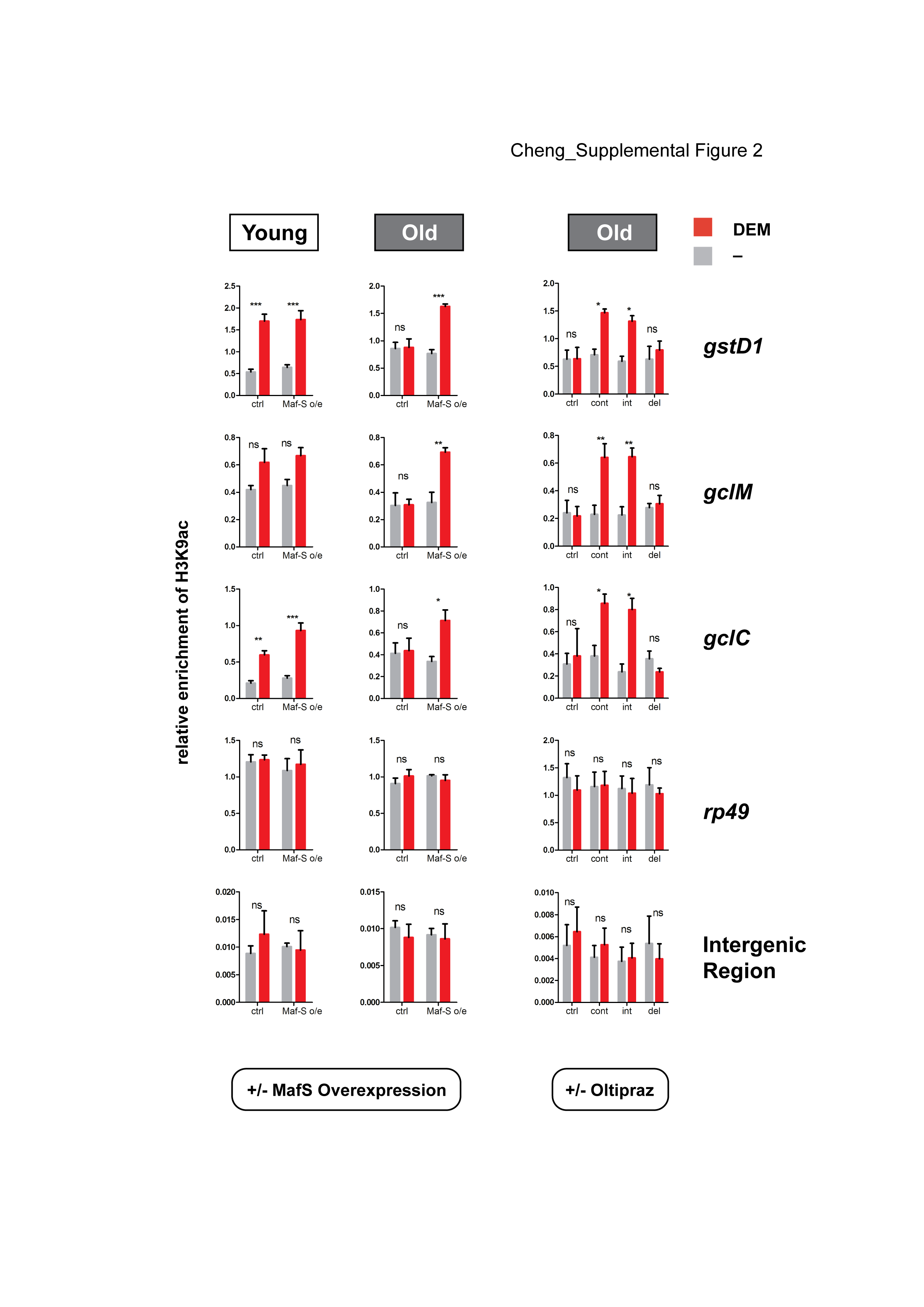

Supplement: Supplementary file 2 — FIGURE S2 [file ACEL-20-e13297-s002.tif]

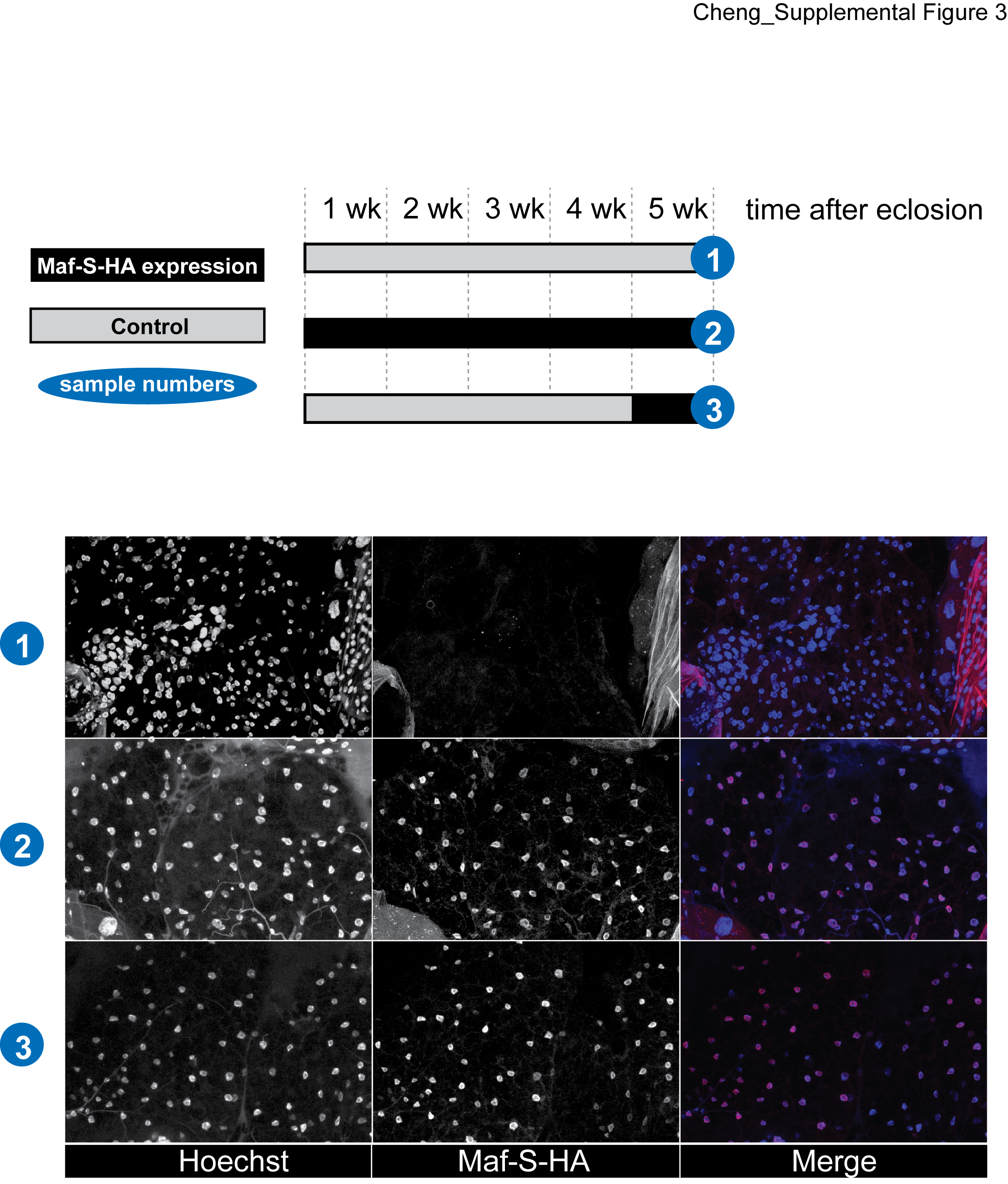

Supplement: Supplementary file 3 — FIGURE S3 [file ACEL-20-e13297-s003.tif]

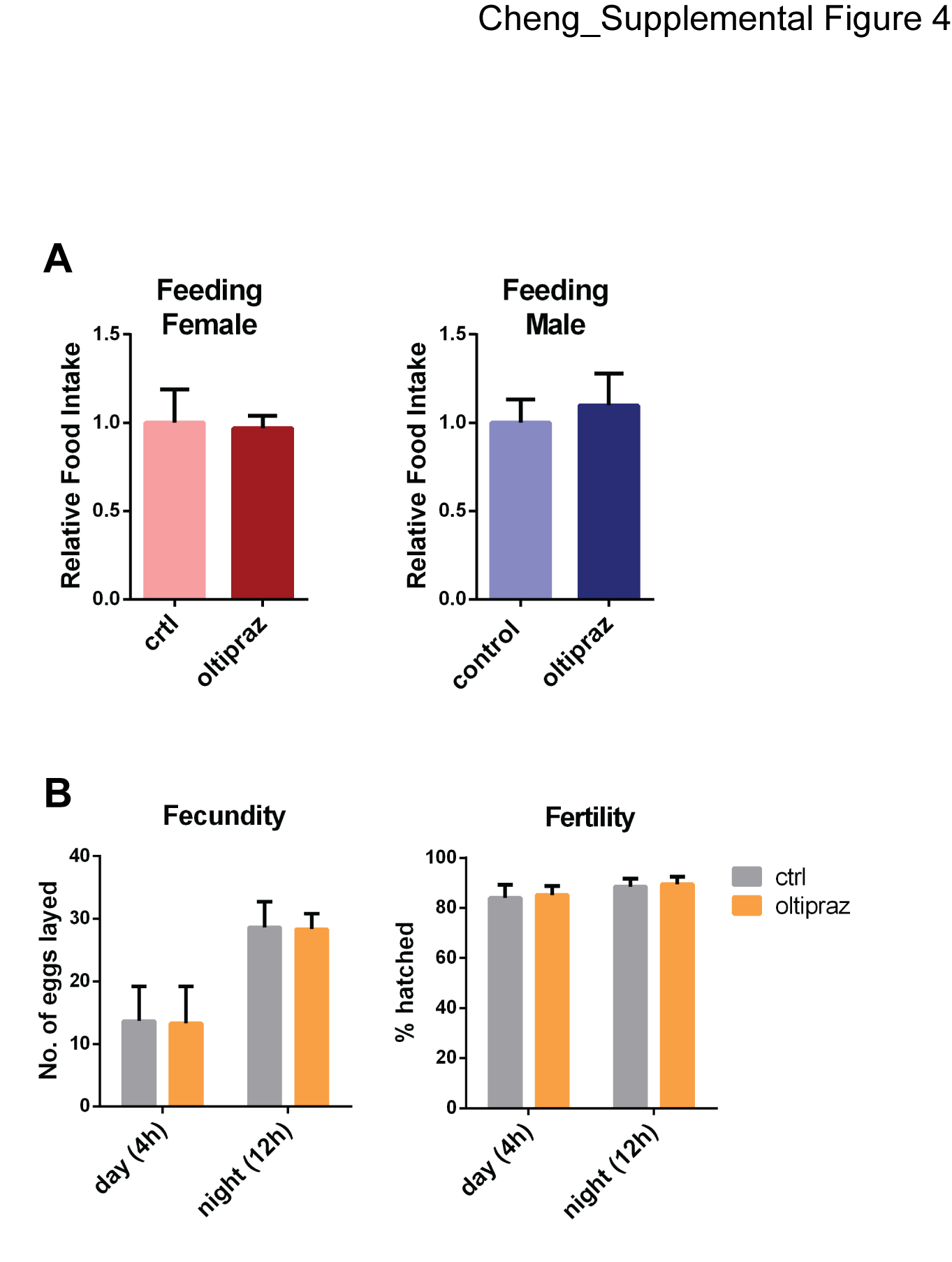

Supplement: Supplementary file 4 — FIGURE S4 [file ACEL-20-e13297-s004.tif]

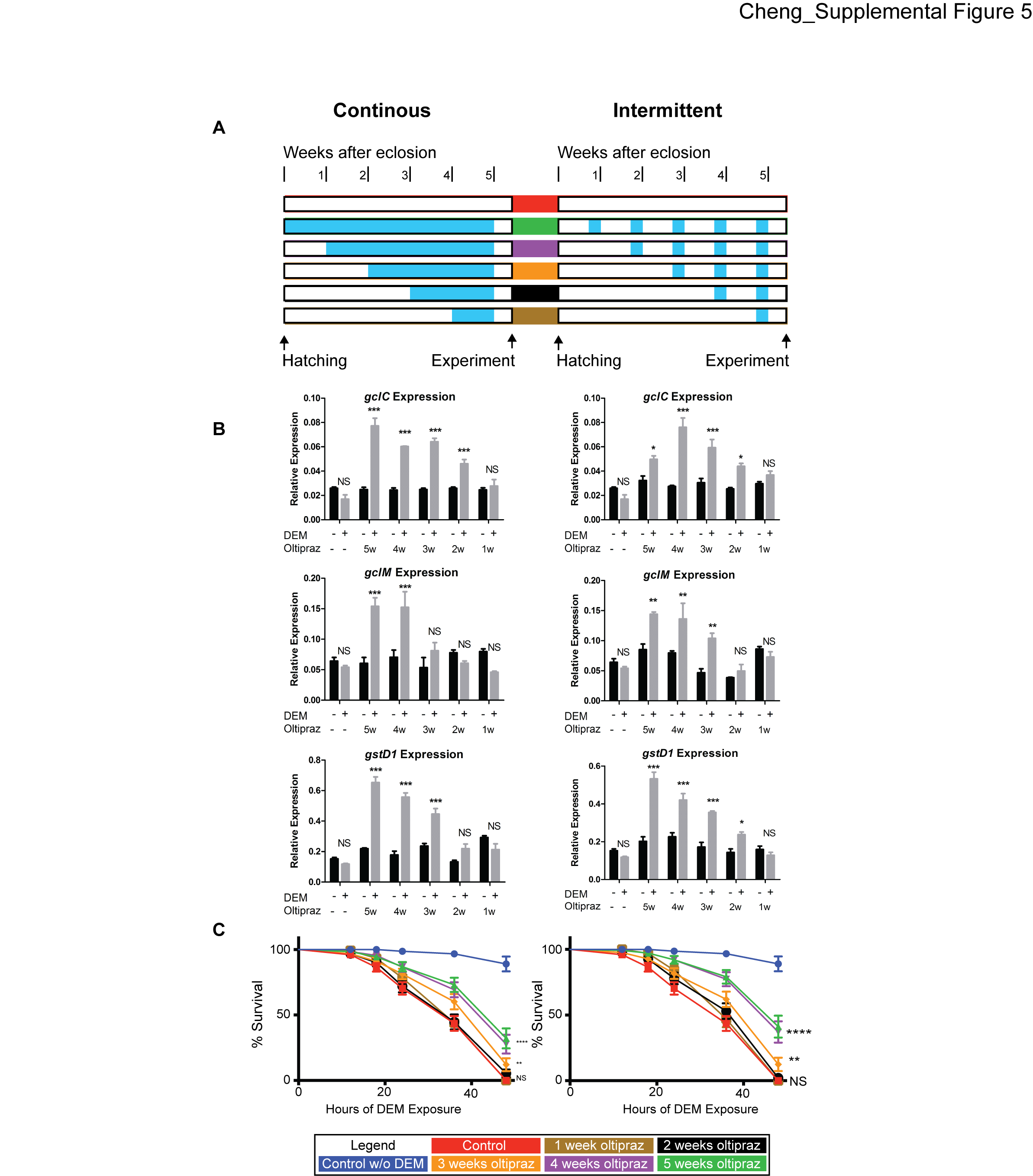

Supplement: Supplementary file 5 — FIGURE S5 [file ACEL-20-e13297-s005.tif]
